# Nitrogen and Biochar Addition Affected Plant Traits and Nitrous Oxide Emission From *Cinnamomum camphora*

**DOI:** 10.3389/fpls.2022.905537

**Published:** 2022-05-10

**Authors:** Congfei Zhu, Handong Luo, Laicong Luo, Kunying Wang, Yi Liao, Shun Zhang, Shenshen Huang, Xiaomin Guo, Ling Zhang

**Affiliations:** ^1^Key Laboratory of Silviculture, Collaborative Innovation Center of Jiangxi Typical Trees Cultivation and Utilization, College of Forestry, Jiangxi Agricultural University, Nanchang, China; ^2^Geological Environment Monitoring Station, Meizhou Natural Resources Bureau, Meizhou, China

**Keywords:** nitrogen addition, leaf traits, plant N_2_O emissions, photosynthesis, structural equation model

## Abstract

Atmospheric nitrous oxide (N_2_O) increase contributes substantially to global climate change due to its large global warming potential. Soil N_2_O emissions have been widely studied, but plants have so far been ignored, even though they are known as an important source of N_2_O. The specific objectives of this study are to (1) reveal the effects of nitrogen and biochar addition on plant functional traits and N_2_O emission of *Cinnamomum camphora* seedlings; (2) find out the possible leaf traits affecting plant N_2_O emissions. The effects of nitrogen and biochar on plant functional traits and N_2_O emissions from plants using *C. camphora* seedlings were investigated. Plant N_2_O emissions, growth, each organ biomass, each organ nutrient allocation, gas exchange parameters, and chlorophyll fluorescence parameters of *C. camphora* seedlings were measured. Further investigation of the relationships between plant N_2_O emission and leaf traits was performed by simple linear regression analysis, principal component analysis (PCA), and structural equation model (SEM). It was found that nitrogen addition profoundly increased cumulative plant N_2_O emissions (+109.25%), which contributed substantially to the atmosphere’s N_2_O budget in forest ecosystems. Plant N_2_O emissions had a strong correlation to leaf traits (leaf TN, *P*_*n*_, *G*_*s*_, *C*_*i*_, *T*r, WUE_*L*_, α, *ETR*_max_, *I*_*k*_, *Fv/Fm*, *Y*(II), and *SPAD*). Structural equation modelling revealed that leaf TN, leaf TP, *P*_*n*_, *C*_*i*_, *T*r, WUE_*L*_, α, *ETR*_max_, and *I*_*k*_ were key traits regulating the effects of plants on N_2_O emissions. These results provide a direction for understanding the mechanism of N_2_O emission from plants and provide a theoretical basis for formulating corresponding emission reduction schemes.

## Introduction

Nitrous oxide (N_2_O) is a potent greenhouse gas with a sustained-flux global warming potentials (SGWPs) 270 times greater than that of carbon dioxide (CO_2_) over a 100-year scale (Neubauer and Megonigal, 2015). N_2_O is also the dominant ozone depleting substance (Ravishankara et al., 2009; Wu et al., 2021).

Soils are considered the major source of N_2_O in forest ecosystems (Chapuis-Lardy et al., 2007). So far, the N_2_O emission between forest and atmosphere is only based on the calculated N_2_O exchange between soil and atmosphere (ButterbachBahl et al., 1997). However, plants have been shown to be involved in N_2_O emissions from soil–plant systems (Chen et al., 1999; Muller, 2003).

Soil microorganisms and plants are two possible sources of N_2_O emissions from plants. One idea is that N_2_O emitted by plants is produced by soil microbes. Plants can transport N_2_O produced by soil to stems and leaves and emit it into the atmosphere (Rusch and Rennenberg, 1998; Pihlatie et al., 2005; Machacova et al., 2013; Diaz-Pines et al., 2016). Another view is that plants produce and emit nitrous oxide, which produce N_2_O during N assimilation processes (Smart and Bloom, 2001). However, the exact mechanism of N_2_O production in plants remains unclear (Goshima et al., 1999; Lenhart et al., 2019).

Methods such as isotopic studies can provide more support for distinguishing the source of nitrous oxide. Based on stable isotope measurement research methods, studies have shown that the dual isotopocule fingerprint of N_2_O released by plants is different from that of all known microbial or chemical processes, indicating that N_2_O released by plants is produced in plant cells (Lenhart et al., 2019). The findings in field conditions challenge the idea that plants may transport N_2_O produced by soil microbes. The site preference (SP) results of soil and plant N_2_O emissions showed that plant cells N_2_O release contributed to total N_2_O emissions under field conditions (Timilsina et al., 2022). Therefore, attention should be paid to the plant N_2_O emissions in forest ecosystems. Understanding the contribution of plants to total N_2_O emissions is also crucial to accurately estimating the global N_2_O budget and identifying possible mitigation options.

China produces about 20 million dead pigs every year, and this number is still rising every year (He et al., 2018). However, there is a lack of research concerning biochar made by dead animals. Pyrolysis of pig carcasses into biochar is an efficient and environmentally friendly option for waste disposal (Yang et al., 2017), while its effects on plant N_2_O emissions and plant functional traits are unclear.

*Cinnamomum camphora* (L.) Presl is a broad-leaved evergreen tree species with important economic value (Chen et al., 2017), whose leaves can be used to extract spices and essential oils (Babu et al., 2003). The intensive N input and leaf-harvesting practice potentially make *C. camphora* plantation soils hot-spots for N_2_O emissions (Zheng et al., 2020), while N_2_O emissions from *C. camphora* plants have not been studied.

Plant photosynthesis is the driving force behind maintaining plant life and biochemical reactions (Hu et al., 2021). The various steps of the photosynthesis process are closely coupled, and changes in any step will affect PSII and cause fluorescence changes. Chlorophyll fluorescence is an effective probe for photosynthesis (Govindjee, and Papageorgiou, 2004). Meanwhile, chlorophyll fluorescence has been found to be an important indicator of photosynthetic energy conversion in chloroplast PSII (Mareckova et al., 2019). Nitrogen (N) and phosphorus (P) are essential nutrients for carbon assimilation in photosynthesis, and the nitrogen and phosphorus content in leaves are closely related to photosynthesis (Mo et al., 2019). Lenhart et al. (2019) established a relationship between N_2_O emission rates and CO_2_ respiration rates, and found that the process of plant-derived emissions may also be related to plant photosynthesis. The release of N_2_O in plants is related to the light and dark reactions of photosynthesis, and the photosynthetic rate and stomatal conductance have a certain relationship with the release of N_2_O in plants (Yang et al., 2005). Studies have shown that plant N_2_O emissions are closely related to their physiological activities. Tree physiological activities include photosynthesis, CO_2_ assimilation, and transpiration. N_2_O transport in transpiration is a mechanism of N_2_O emission in plants. In addition, N_2_O is also produced in plant tissues during nitrate assimilation, which is closely related to photosynthesis (Machacova et al., 2019). As mentioned above, existing studies have greatly advanced our understanding of the correlation between N content, P content and physiological activity in leaves. However, specific studies on the relationship between plant N_2_O emissions and these leaf traits under nitrogen and biochar addition are still lacking.

In this study, we used the closed box method and *C. camphora* seedlings to monitor the changes in plant N_2_O emissions as affected by N and biochar addition. In addition, we also investigated the deep relationship between plant N_2_O emissions and the functional traits of *C. camphora* seedlings. The objectives of this study were to:

(1) Study nitrogen and dead pig-derived biochar effects on plant N_2_O emissions of *C. camphora*; (2) explore plant traits and physiological parameters that influence plant N_2_O emissions.

## Materials and Methods

### Pot Experiment Design

This study was conducted in Jiangxi Agricultural University, Jiangxi, China (28°46′05′′N, 115°50′22′′E) from November 2017 (seeds collection) to November 2018 (seedling harvest). According to the Chinese classification system of Quaternary Red Clay, the soil in the pot experiment is classified as typical red soil. Seeds of *C. camphora* were planted in January 2018. After seeds germinated in early April, *C. camphora* seedlings were transplanted into a plastic pot filled with 2 kg soil passed through a 2 mm sieve. At the end of July 2018 (growth period for *C. camphora*), seedlings of the same size for experimentation were selected. The growth period of *C. camphora* in the experiment was consistent with that in this area. In this pot experiment, eight treatments with four replicates were carried out.

A full factorial randomized design with four N (N_0_, 0 mg N kg^–1^ dry soil; N_1_, 100 mg N kg^–1^ dry soil; N_2_, 200 mg N kg^–1^ dry soil; and N_3_, 300 mg N kg^–1^ dry soil) and two pig carcass biochar levels (BC_0_, control; and BC_1_, 1% pig biochar, w/w) (Chen et al., 2020) was employed. Nitrogen addition was performed by spraying the same volume of urea [CO(NH_2_)_2_] solution (1 g N L^–1^, 2 g N L^–1^ and 3 g N L^–1^) and was applied twice on August 2 and September 1, 2018. The biochar was derived from pig carcasses (Huzhou Industrial and Medical Waste Treatment Center, Zhejiang, China) and was ground to pass through a 2 mm sieve before application. Biochar was dissolved in water and applied to the soil of *C. camphora* seedlings on 2 August 2018. Both soil and biochar characteristics are shown in Supplementary Table 1. See Table 1 for a list of measured *C. camphora* plant traits with their abbreviations and units.

**TABLE 1:** List of *Cinnamomum camphora* plant traits measured, their abbreviations and units.

Abbreviation	Plant trait	Unit
PH	Plant height	cm
GD	Ground diameter	mm
LN	Leaf number	

LM	Leaf mass	g
SM	Stem mass	g
RM	Root mass	g
TM	Total mass	g
R:S	Root shoot ratio	

Leaf TN	Leaf total nitrogen content	g kg^–1^
Leaf TP	Leaf total phosphorus content	g kg^–1^
Leaf TK	Leaf total potassium content	g kg^–1^
Stem TN	Stem total nitrogen content	g kg^–1^
Stem TP	Stem total phosphorus content	g kg^–1^
Stem TK	Stem total potassium content	g kg^–1^
Root TN	Root total nitrogen content	g kg^–1^
Root TP	Root total phosphorus content	g kg^–1^
Root TK	Root total potassium content	g kg^–1^

*P*_*n*_	Net photosynthetic rate	μmol CO_2_ m^–2^ s^–1^
*G*_*s*_	Stomatal conductance	mol H_2_O m^–2^ s^–1^
*C*_*i*_	Intercellular CO_2_ concentration	μmol CO_2_ mol^–1^
*T*r	Transpiration rate	mmol H_2_O m^–2^ s^–1^
WUE_*L*_	Leaf instantaneous water use efficiency	μmol CO_2_ mmol H_2_O^–1^

α	The initial slope of the fast light response curve	Electrons photons^–1^
*ETR*_*max*_	Potential maximum relative electron transfer rate	μmol m^–2^ s^–1^
*I*_*k*_	Half full and light intensity	μmol m^–2^ s^–1^
*F_*v*_/F_*m*_*	Maximum quantum yield of photosystem II	
*Y(II)*	Effective quantum yield of photosystem II	
*SPAD*	Relative chlorophyll content	

### Measurement of Plant N_2_O Emissions

**FIGURE 1:**
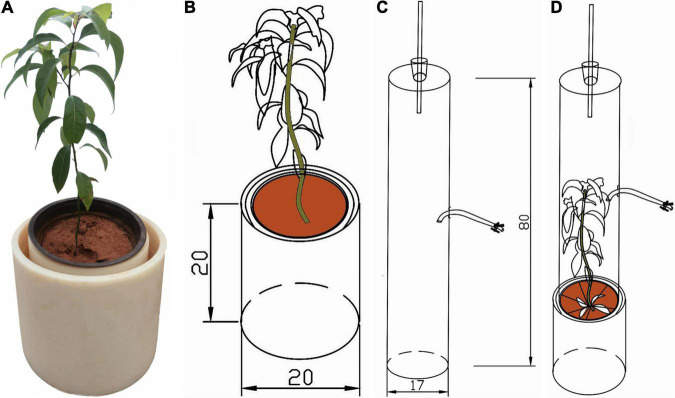
Schematic of the chamber designed to measure plant N_2_O emissions from *Cinnamomum camphora*. **(A)** Plant + collar groove test diagram, **(B)** plant + collar groove schematic diagram, **(C)** sampling chamber, **(D)** closed transparent assembled chamber.

Plant N_2_O fluxes were measured by using a closed transparent chamber (diameter × height = 17 cm × 80 cm) (Figure 1). The sampling chamber was a cylinder made of PVC tube. On the top of the cylinder, there was a small hole, into which a thermometer with a rubber plug was placed. Then they inserted a rubber hose in the middle of the barrel, which was connected by a three-way valve on the outside of the hose (Figure 1). Aluminum foam was used to cover the outer surface to reduce temperature change during sampling. Before gas collection, distilled water was injected into the collar groove with a syringe for airtight sealing (Figure 1). Overall, this experiment consisted of 32 pots with four replicates and 14 times measurements of plant N_2_O emissions (4N × 2 biochar × 4 replicates × 14 times). When plant N_2_O emissions were measured, soil was wrapped by plastic bag (Figure 1). During the gas collection, the chamber was sealed to ensure it was airtight. A 60 ml syringe was used to collect gas samples at 0, 10, 20, and 30 min after the chambers were closed. Gas samples were immediately transferred into a 100 mL aluminum foil gas bag. Gas samples were collected between 9:00 and 11:00 (China Standard Time). The collected gas samples were immediately taken back to the laboratory for concentration determination. N_2_O concentrations were determined within 24 h after sampling using a gas chromatograph (Agilent 7890B, Santa Clara, CA, United States) equipped with an electron capture detector (ECD). Because most of the plant N_2_O emissions are emitted by leaves (Li and Chen, 1993), the plant N_2_O emissions of *C. camphora* were calculated based on leaf area. After each gas collection, leaf area was measured by a hand-held laser blade area meter (CID, CI-203, America). The leaf area was measured 14 times. The plant N_2_O emissions were shown as μg m^–2^ leaves h^–1^ (Pihlatie et al., 2005). Plant nitrous oxide fluxes (*F*, μg m^–2^ leaves h^–1^) were calculated by the following equation (Bowatte et al., 2014):


(1) $$F=P\times V\times \frac{\Delta \,c}{\Delta \,t}\times \frac{1}{\mathrm{RT}}\times M\times \frac{1}{s}$$

where *P* is the standard atmospheric pressure (Pa); *V* and *S* are the cylindrical chamber volume (m^3^) and seeding leaf area (m^2^); Δc/Δt means the rate of N_2_O (ppb) concentration change with time based on linear regressions; *R* indicates the universal gas constant; *T* is the absolute air temperature (K) when the gas sample was aspirated.

Cumulative plant N_2_O emissions (*E*, μg m^–2^) were calculated by Abalos et al. (2018):


$$E=\sum_{i=1}^{n}\frac{\left(F_{i}+F_{i+1}\right)}{2}\times \left(t_{i+1}-t_{i}\right)\times 24$$

where *E* is the cumulative plant N_2_O emissions (μg m^–2^ leaves); *F* and *i* are the plant N_2_O emission rates (μg m^–2^ leaves h^–1^) and *i* th gas collection, respectively; (*t_*i*+1_*−*t*_*i*_) refers to the interval time of two gas collection; *n* is the total number of gas collection times. Fluxes of N_2_O were measured 14 times from 22 July 2018 to 17 November 2018 at days 1, 11, 13, 16, 19, 35, 43, 46, 49, 57, 74, 88, 106, 119. During the experiment, *C. camphora* seedlings were regularly irrigated with equal amounts of distilled water. During the gas collection periods, *C. camphora* seedlings were irrigated 3 days in advance.

### Measurement of Leaf Gas Exchange and Chlorophyll Fluorescence Parameters

Before harvesting, the gas exchange parameters, chlorophyll fluorescence parameters, and relative chlorophyll content of seedlings were measured. In this pot experiment, 32 pots were carried out for measurement. The net photosynthetic rate *P*_*n*_ (μmol CO_2_ m^–2^ s^–1^), stomatal conductance *G*_*s*_ (mol H_2_O m^–2^ s^–1^), intercellular CO_2_ concentration *C*_*i*_ (μmol CO_2_ mol^–1^) and transpiration rate *Tr* (mmol H_2_O m^–2^ s^–1^) of the fourth fully expanded leaf were measured by a LI-6400XT photosynthesis system (LI-COR, Lincoln, NE, United States) between 8:30 and 11:30 a.m. on a sunny day. For each *C. camphora* seedling, three fully expanded leaves were selected for the photosynthetic measurement. The parameters were set as follows: the measured light was 1,000 μmol m^–2^ s^–1^, the CO_2_ concentration in the leaf chamber was 400 μmol mol^–1^, the temperature in the leaf chamber was 25°C, and the air flow rate was 500 ml min^–1^ (Gong et al., 2022). Leaf instantaneous water use efficiency WUE_*L*_ (μmol CO_2_ mmol H_2_O^–1^) was calculated as follows: WUEL = *P*_*n*_/*Tr* (Fang et al., 2018).

For each *C. camphora* seedling, three representative leaves were selected for the chlorophyll fluorescence measurement. Chlorophyll fluorescence parameters of representative leaves were measured by a Pulse-Amplitude-Modulation (PAM) fluorometer (PAM 2500, Walz GmbH, Nuremberg, Germany). The minimum (*F*_0_) and maximum (*F*_*m*_) fluorescence were recorded after sufficient dark adaptation (at least 30 min) of the sample with a dark adaptation clip. The leaves were given saturated pulsed light (2,000 μmol m^–2^ s^–1^) for 3 s and actinic light (300 μmol m^–2^ s^–1^) for 10 min. The chlorophyll fluorescence peak was recorded as *F_*m*_′*, and before the saturation pulse was turned off, the recorded fluorescence value was *F*. The maximum quantum yield of photosystem II, *F*_*v*_/*F*_*m*_ = (*F*_*m*_ −*F*_0_)/*F*_*m*_. The effective quantum yield of photosystem II, *Y*(II) = Δ*F*/*F_*m*_′* = (*F_*m*_′*-*F*)/*F_*m*_′* (Kitajima and Butler, 1975; Genty et al., 1989). At night, after the samples had passed sufficient dark adaptation, the actinic light with light intensities of 2, 6, 31, 101, 141, 271, 474, 785, 1,160, and 1,663 μmol m^–2^ s^–1^ was turned on in turn. The irradiation time of actinic light for each intensity was 10 s. Using the Pam Win-3 software, the fast light response curve was fitted with the formula (Eilers and Peeters, 1988). α, initial slope of fast light curve (electrons photons^–1^); *ETR*_*max*_, potential maximum relative electron transfer rate (μmol m^–2^ s^–1^); *I*_*k*_, half full and light intensity (μmol m^–2^ s^–1^) (Ralph and Gademann, 2005). The *SPAD*-502 Plus (Konicaminolta, Japan) was used to determine the relative chlorophyll content (*SPAD*). For each *C. camphora* seedling, four fully expanded leaves were selected for measurement.

### Measurement of Seedling Biomass and Nutrient

At the end of the study, whole plant seedlings were harvested in November 2018. In this pot experiment, 32 *C. camphora* seedlings were harvested. All seedlings were gently processed by removing soil. Then seedling height, ground diameter, leaf number, and leaf area were measured and recorded. The leaves, stems, and roots of seedlings were cleaned with pure water and dried to a constant weight, which was weighed separately. The aboveground mass (leaf mass+stem mass), underground mass (root mass), total mass (underground mass+aboveground mass) and root-shoot ratio (R: S = underground mass: aboveground mass) could be obtained by calculation (Zhang et al., 2013; Deng et al., 2019). After the above data were recorded, the roots, stems and leaves of seedlings were crushed to pass through 0.149 mm sieve for determination of N, P, and K. The total phosphorus content was determined by molybdenum blue colorimetry, the total nitrogen content was determined by indophenol blue colorimetry after removing part of the digestion liquid and adjusting to neutral pH, and the total potassium in the digestion liquid was determined by flame photometry (Chen et al., 2022).

### Statistical Analyses

Analysis of variance (ANOVA) was used to examine the dependence of plant N_2_O emission rate and leaf area as affected by N and biochar and their interactions as fixed effects and sampling time as random effects (Xu et al., 2020). Two-way ANOVA was used to examine the dependence of cumulative plant N_2_O emissions and plant trait parameters on N, biochar, and their interactions. Tukey *post-hoc* tests were used to examine differences among means with significant results. Pearson correlation analysis to explore the linear relationship between leaf traits and plant N_2_O emissions (Chen et al., 2015). Origin 2021 was used to conduct principal component analysis (PCA) to analyze leaf traits and determine the main functional traits of N_2_O emission in plants (Li et al., 2022). AMOS 26.0 (IBM Corp, Armonk, NY, United States) was employed to perform structural equation modelling (SEM) to detect the influences among the variables. The model was constructed using our hypotheses about plant functional traits affecting plant N_2_O emissions. The quality of the SEM model was assessed by using the chi-square goodness-of-fit statistic χ^2^/*df*, Akaike’s information criterion (AIC), the Bayesian information criteria (BIC), the root mean square error of approximation value (RMSEA), the comparative fit index (CFI), and the standardized root mean square residual (SRMR) (Grace, 2006). We used JMP 9.0 (Cary, NC, United States) for data analysis.

## Results

### Plant Traits as Effected by N and Biochar Addition

Plant height, ground diameter, and leaf number differed significantly among different levels of N and between two different levels of biochar addition (*P* < 0.01; Table 2). The same was true for leaf mass, stem mass, root mass, and total mass. These differences could be ascribed to the absorption and utilization of different nutrients by *C. camphora*. Plant height, ground diameter, leaf number, and each organ mass showed an increasing trend with the addition of N supplemental level regardless of adding biochar or not. However, root shoot ratio had no significant difference among different levels of N and between two different levels of biochar addition (Supplementary Figure 1).

**TABLE 2:** Dependence of plant growth and biomass on N (0, 100, 200, and 300 mg N kg^–1^ dry soil) and biochar addition (control and biochar addition) and their interactions in two-way ANOVAs.

Variables	Nitrogen			Biochar			Nitrogen × Biochar		
	DF	*F*	*P*	DF	*F*	*P*	DF	*F*	*P*
Plant height	3	41.5	**<0.0001**	1	25.6	** < 0.0001**	3	1.6	0.229
Ground diameter	3	93.8	**<0.0001**	1	55.1	** < 0.0001**	3	0.8	0.494
Leaf number	3	50.0	**<0.0001**	1	18.4	**0.0003**	3	1.6	0.231
Leaf mass	3	57.0	**<0.0001**	1	7.9	**0.011**	3	0.61	0.617
Stem mass	3	55.5	**<0.0001**	1	17.3	**0.0004**	3	1.0	0.401
Root mass	3	100.2	**<0.0001**	1	15.2	**0.001**	3	0.2	0.897
Total mass	3	136.2	**<0.0001**	1	26.0	** < 0.0001**	3	0.9	0.463
Root shoot ratio	3	0.8	0.52	1	0.03	0.867	3	0.4	0.761

*Significant results are shown in bold.*

Leaf TN, stem TN, and root TN differed significantly between levels of N addition (*P* < 0.0001; Table 3). Leaf, stem and root TN content of all treatments ranged from 11.73 g kg^–1^ to 17.70 g kg^–1^,7.84 g kg^–1^ to 14.84 g kg^–1^, and 9.76 g kg^–1^ to 14.85 g kg^–1^ (Supplementary Figure 2). Leaf TP, TK, stem TP, TK, root TP, and TK differed significantly between levels of biochar addition (*P* < 0.01; Table 3). Compared with control, biochar increased TP content in leaves and roots (+20.63 and +14.62%), respectively (*P* < 0.05; Supplementary Figure 2). Biochar increased TK content in leaves, stems, and roots (+19.78, 32.44, 33.00%), respectively (*P* < 0.05; Supplementary Figure 2).

**TABLE 3:** Dependence of TN, TP, and TK in leaf, stem and root on N (0,100,200 and 300 mg N kg^–1^ dry soil) and biochar addition (control and biochar addition) and their interactions in two-way ANOVAs.

Variables	Nitrogen			Biochar			Nitrogen × Biochar		
	DF	*F*	*P*	DF	*F*	*P*	DF	*F*	*P*
Leaf TN	3	29.1	** < 0.0001**	1	0.03	0.854	3	1.3	0.287
Leaf TP	3	0.01	0.998	1	26.2	** < 0.0001**	3	0.4	0.742
Leaf TK	3	0.5	0.686	1	14.1	**0.002**	3	1.7	0.226
Stem TN	3	85.8	** < 0.0001**	1	0.2	0.686	3	1.1	0.375
Stem TP	3	0.1	0.976	1	5.3	**0.03**	3	0.3	0.793
Stem TK	3	0.1	0.964	1	37.0	** < 0.0001**	3	1.9	0.162
Root TN	3	17.6	** < 0.0001**	1	0.5	0.508	3	0.3	0.811
Root TP	3	1.4	0.271	1	21.6	**0.0001**	3	1.7	0.187
Root TK	3	1.8	0.181	1	353.0	**0.0001**	3	2.9	0.056

*Significant results are shown in bold. TN, total nitrogen; TP, total phosphorus; TK, total potassium.*

Gas exchange parameters and chlorophyll fluorescence parameters had significant responses to nitrogen addition. At the same time, biochar affects most physiological indicators (*P* < 0.01; Table 4). The mean values of *P*_*n*_, *G*_*s*_, *T*r, and WUE_*L*_ became larger as N addition increased (Supplementary Figure 3). Nitrogen and biochar had significant interaction on *P*_*n*_ and *T*r (*P* < 0.001; Table 4 and Supplementary Figure 3). For all nitrogen and biochar levels, the maximum mean values of *P*_*n*_ and *T*r were 7.82 μmol CO_2_ m^–2^ s^–1^ and 1.99 mmol H_2_O m^–2^ s^–1^ as compared to control (Supplementary Figure 3). α, *ETR*_*max*_, *I*_*k*_, *Fv/Fm*, *Y*(II), and *SPAD* differed significantly among different levels in N and between two different levels in biochar addition (*P* < 0.05; Table 4 and Supplementary Figure 4). The maximum mean values of α, *ETR*_*max*_, *I*_*k*_, *Fv/Fm*, *Y*(II), and *SPAD* are 0.18 electrons photons^–1^, 35.08 μmol m^–2^ s^–1^, 199.90 μmol m^–2^ s^–1^, 0.84, 0.36, and 42.73 (Supplementary Figure 4).

**TABLE 4:** Dependence of plant physiological parameters and plant N_2_O emissions on N (0, 100, 200, and 300 mg N kg^–1^ dry soil) and biochar addition (control and biochar addition) and their interactions in two-way ANOVAs.

Variables	Nitrogen			Biochar			Nitrogen × Biochar		
	DF	*F*	*P*	DF	*F*	*P*	DF	*F*	*P*
*P*_*n*_	3	238.2	** < 0.0001**	1	59.0	** < 0.0001**	3	9.2	**0.0003**
*G*_*s*_	3	31.3	** < 0.0001**	1	6.4	**0.018**	3	1.0	0.4
*C*_*i*_	3	5.3	**0.006**	1	0.4	0.528	3	2.5	0.087
*T*r	3	86.2	** < 0.0001**	1	60.2	** < 0.0001**	3	8.5	**0.0005**
WUE_*L*_	3	35.9	** < 0.0001**	1	0.3	0.615	3	2.4	0.09
α	3	8.5	**0.0005**	1	6.4	**0.018**	3	0.6	0.626
*ETR*_*max*_	3	15.7	** < 0.0001**	1	6.5	**0.017**	3	0.05	0.984
*I*_*k*_	3	10.2	**0.0002**	1	2.7	0.116	3	0.2	0.903
*Fv/Fm*	3	35.9	** < 0.0001**	1	14.1	**0.001**	3	2.9	0.055
*Y*(II)	3	25.5	** < 0.0001**	1	4.1	0.054	3	0.5	0.656
*SPAD*	3	48.0	** < 0.0001**	1	8.8	**0.007**	3	1.0	0.397
C-plant N_2_O	3	255.2	** < 0.0001**	1	0.9	0.353	3	1.9	0.151

*Significant results are shown in bold. P_n_, net photosynthetic rate; G_s_, stomatal conductance; C_i_, intercellular CO_2_ concentration; Tr, transpiration rate; WUE_L_, leaf instantaneous water use efficiency; α, the initial slope of the fast light response curve; ETR_max_, potential maximum relative electron transfer rate; I_k_, half full and light intensity; Fv/Fm, maximum quantum yield of photosystem II; Y(II), effective quantum yield of photosystem II; C-plant N_2_O, cumulative plant N_2_O emissions.*

### Relationships of Plant N_2_O Emissions and Leaf Traits

**FIGURE 2:**
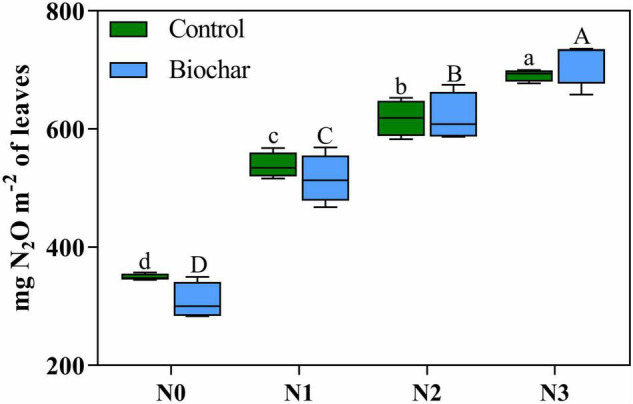
Cumulative plant N_2_O emissions in biochar and control conditions at different nitrogen levels. Significant differences between nitrogen additions are indicated by a small letter (control, BC_0_), or a capital letter (biochar, BC_1_). There is no difference between biochar and control conditions. Tukey *post-hoc* tests were used to determine significance among levels of nitrogen.

**FIGURE 3:**
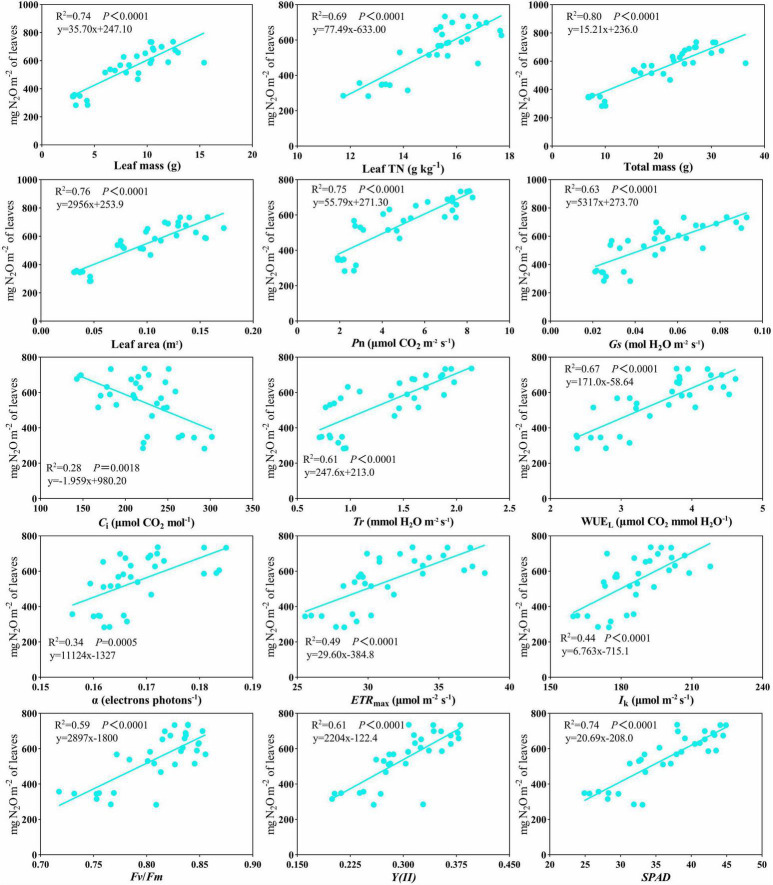
Scatterplot of the relationship of plant N_2_O emissions versus leaf mass, leaf TN, total mass, leaf area, *P*_*n*_, *G*_*s*_, *C*_*i*_, *T*r, WUE_*L*_, α, *ETR*_*max*_, *I*_*k*_, *Fv/Fm*, *Y*(II), and *SPAD*. Lines indicate significant relations.

Plant N_2_O emissions were significantly affected by N addition and increased with N addition levels (Table 4 and Figure 2). No significant differences in cumulative plant N_2_O emissions were recorded between biochar and control conditions (Figure 2). In addition, the leaf area of *C. camphora* seedlings was also increased by N and biochar during the study based on the dynamics (Supplementary Figure 5). Plant N_2_O emission rates were significantly affected by N addition and increased with N addition levels (Supplementary Figure 6). However, the plant N_2_O emission rates had no significant difference between the two different levels of biochar addition (Supplementary Figure 6). Plant N_2_O emissions were positively correlated with leaf mass (Figure 3, R^2^ = 0.74, *P* < 0.0001), leaf TN (R^2^ = 0.69, *P* < 0.0001), total mass (R^2^ = 0.80, *P* < 0.0001), leaf area (R^2^ = 0.76, *P* < 0.0001), *P*_*n*_ (R^2^ = 0.75, *P* < 0.0001), *G*_*s*_ (R^2^ = 0.63, *P* < 0.0001), *T*r (R^2^ = 0.61, *P* < 0.0001), WUE_*L*_ (R^2^ = 0.67, *P* < 0.0001), α (R^2^ = 0.34, *P* = 0.0005), *ETR*_*max*_ (R^2^ = 0.49, *P* < 0.0001), *I*_*k*_ (R^2^ = 0.44, *P* < 0.0001), *Fv/Fm* (R^2^ = 0.59, *P* < 0.0001), *Y*(II) (R^2^ = 0.61, *P* < 0.0001), and *SPAD* (Figure 3, R^2^ = 0.74, *P* < 0.0001). However, it was negatively correlated with *C*_*i*_ (Figure 3, R^2^ = 0.28, *P* = 0.0018).

**FIGURE 4:**
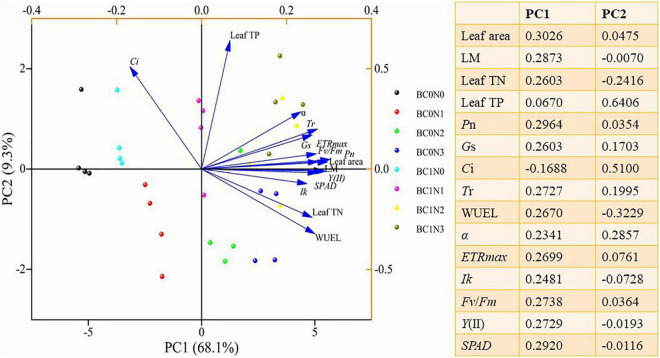
Principal component analysis (PCA) plots of plant functional traits under biochar and control conditions (BC_0_, control; BC_1_, biochar addition) at different nitrogen (0 mg N kg^–1^ dry soil, N_0_; 100 mg N kg^–1^ dry soil, N_1_; 200 mg N kg^–1^ dry soil, N_2_; 300 mg N kg^–1^ dry soil, N_3_) levels.

To further clarify the relationship between leaf traits and plant N_2_O emissions, a principal component analysis (PCA) was performed using leaf area, leaf biomass, leaf TN, leaf TP, and physiological indicators (Figure 4). PC1 and PC2 accounted for 68.10 and 9.30% of the investigated variation, respectively. Leaf area, LM, leaf TN, *P*_*n*_, *G*s, *T*r, WUE_*L*_, α, *ETR*_*max*_, *I*_*k*_, *F*v/*F*m, *Y*(II), and *SPAD* were more influenced by PC1, while leaf TP and Ci were more influenced by PC2. At the same time, each treatment has a good degree of differentiation.

### Relationships Among Functional Traits and Mechanisms Linking Leaf Traits and Plant N_2_O Emissions

**FIGURE 5:**
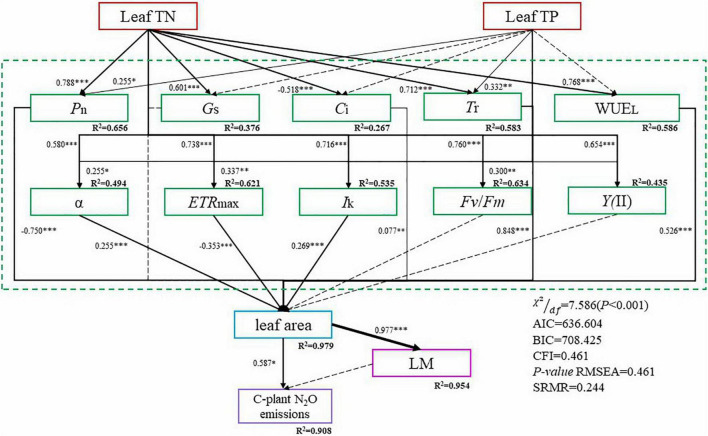
Structural equation models (SEM) were used to evaluate the effects of leaf traits on plant N_2_O emissions. Dashed lines represent non-significant relationships, solid lines represent significant relationships (*P < 0.05; ^**^P < 0.01; ^***^P < 0.001). Numbers next to the arrows represent the (positive or negative) standardized path coefficients. R^2^ values represent the amount of variance interpreted by the model for the response variables.

The results are exhibited in Figure 5. As for the model fit indices, which are shown in the lower right, all indices indicate that our hypothesized model was acceptable [χ^2^/*df* = 7.586 (*P* < 0.001), AIC = 636.604; BIC = 708.425; CFI = 0.461; RMSEA = 0.461; SRMR = 0.244]. Additionally, the modification indices were low, indicating that our model could not be further improved by adding omitted relationships.

The results revealed that leaf traits do affect plant N_2_O emissions in a direct or indirect way. As a multi-staged path model, we could read from the graph that leaf TN significantly affected *P*_*n*_, *G*_*s*_, *C*_*i*_, *T*r, WUE_*L*_, α, *ETR*_max_, *I*k, *Fv/Fm*, *Y*(II). Among them, Leaf TN would significantly increase the *P*_*n*_, *G*_*s*_, *T*r, WUE_*L*_, α, *ETR*max, *I*k, *Fv/Fm*, *Y*(II), while significantly decreasing the *C*_*i*_. For Leaf TP, it has a significant influence on *P*_*n*_, *T*r, α, *ETR*max, and *Fv/Fm*. Leaf TP would significantly increase the above indicators. *P*_*n*_, *C*_*i*_, *T*r, WUE_*L*_, α, *ETR*max, and *I*k all had a significant impact on leaf area. *P*_*n*_ and *ETR*max would significantly decrease the leaf area, while the rest would significantly increase the leaf area. Leaf area influences leaf mass and cumulative plant N_2_O emissions significantly and positively, while leaf mass doesn’t have a significant influence on cumulative plant N_2_O emissions. The relationship between the remaining variables was not significant, but improved the model fitting.

## Discussion

### Plant Traits and N_2_O Emissions as Effected by N and Biochar Addition

Nitrogen is an essential macronutrient and plays an important role in plant growth and development (Galloway et al., 2002). Biochar has positive effects on plant growth (Dong et al., 2015; Purakayastha et al., 2019). Plant biomass was increased by animal carcass-derived biochar addition (Chen et al., 2020). These results support that N and biochar addition significantly increased the growth indexes (plant height, ground diameter, leaf number, biomass, etc.) of *C. camphora* seedlings (Supplementary Figure 1). In this study, both nitrogen and biochar increased leaf mass, stem mass, and root mass (Supplementary Figure 1), indicating that nitrogen and biochar’s effects on root-shoot ratio might have been offset by their effects on aboveground biomass and underground biomass. Recent studies have shown that net photosynthesis rate (*P*_*n*_) and transpiration rate (*T*_*r*_) increased by 19 and 40% in the biochar treatments, respectively, compared to control (Zulfiqar et al., 2021). Biochar significantly increased net photosynthetic rate, transpiration rate, stomatal conductance, and water use efficiency during the plant growth period, relative to control (Wang et al., 2021). Chlorophyll fluorescence parameters can effectively reflect the absorption, utilization, and transformation of light energy. *Fv/Fm* stands for the maximum quantum yield of PSII, which can reflect the potential maximum light energy conversion efficiency of plants. *Y*(II) represents the actual photosynthetic quantum yield of PSII, which can reflect the current actual light energy conversion efficiency of photosynthetic organs (Baker, 2008). Research has shown that biochar has great potential in improving chlorophyll fluorescence (Wang et al., 2021). That’s probably because biochar has the effect of increasing the chlorophyll content of leaves (Feng et al., 2021), which can ensure the synthesis of various enzymes and electron transporters in the process of carbon assimilation in photosynthesis, thereby improving the function of leaf photosynthesis (Hou et al., 2021).

Plant N_2_O emissions were affected by N but not by biochar addition (Figure 2). With the increase of N addition levels, plant N_2_O emissions increased consistently (Figure 2). Since leaves have been proved to be the main place emitting N_2_O, factors influencing leaf area might also impact plant N_2_O emissions. In this study, both biochar and N increased leaf area (Supplementary Figure 5). Biochar had no effect on plant N_2_O emissions (Table 4, Figure 2, and Supplementary Figure 6), indicating biochar’s effects on plant N_2_O emissions might have been offset by its effects on N_2_O production and leaf area. Indeed, plant N_2_O emissions may be a phenomenon emitting N_2_O that was produced by plants or emitting soil originated N_2_O to atmosphere (Chang et al., 1998). Even though it was not studied here, plant production of N_2_O by *C. camphora* seedlings could be possible since a ^15^N isotopic labelling study revealed that N_2_O released from wheat leaves originated from NO_3_^–^ assimilation by wheat plants instead of microorganisms in the rhizosphere (Smart and Bloom, 2001). Plant-mediated N_2_O emissions have been reported in agricultural, wetland, and forest plants (Pihlatie et al., 2005). N_2_O produced in soil could also be transported to atmosphere by transpiration streams of plants (Pihlatie et al., 2005). In our study, biochar mitigated soil N_2_O emissions while increasing leaf area (unpublished data). Therefore, N_2_O produced in the soil and emitted by plants could not be the important source of N_2_O emissions by *C. camphora* seedlings. However, N could increase plant N_2_O emissions by providing more N substrate readily available for plant, increasing NO_3_^–^ and NO_2_^–^ assimilation by plant roots and leaves, respectively, which will directly enhance plant N_2_O emissions (Smart and Bloom, 2001; Pihlatie et al., 2005). The cumulative N_2_O emissions of plants increase with the increase of nitrogen levels (Figure 2). Leaf nitrogen content is directly proportional to the N_2_O emission of plants (Figure 3). The rate of N_2_O release by plants is related to the utilization of nitrogen by plants, which may be due to the release of nitrogen absorbed by plants, especially NO_3_^–^. After being reduced to NO_2_^–^ by nitrate reductase (NR), part of it is further reduced to N_2_O and released. NR is a substrate inducible enzyme. When nitrogen is added to the soil, plants grow well, have high NR activity, and produce more N_2_O. With the increase in nitrogen levels, plants grow fast and produce a large amount of N_2_O (Li and Chen, 1993). Soybean and maize seedlings can release N_2_O by themselves, and the N_2_O release is related to the amount of nitrogen and phosphorus application. The direct emission of N_2_O by plants may be a physiological defence of plants to avoid excessive accumulation of NO_3_^–^ under the restriction of other growth factors, which will lead to a decline in the nitrogen use efficiency of plants (Chen et al., 1995).

### Relationships Between Plant N_2_O Emissions and Various Leaf Traits

Our results provide powerful data for understanding the N_2_O emission patterns of seedlings during their growing period (Supplementary Figure 6). Importantly, we found a close relationship between leaf traits and plant N_2_O emissions (Figures 3–5). Our continuous emission measurement method is an effective improvement over the traditional indoor study because it overcomes the influence of the device during seedling growth and better reflects the temporal variation of N_2_O emission flux (Supplementary Figure 6). Therefore, our method is suitable for measuring plant N_2_O emissions during seedling growth and can be used to explore deeper association analysis.

Leaf photosynthetic capacity is related to leaf N concentration (Figures 4, 5). N-rich compounds [ribulose-1,5-bisphosphate carboxylase/oxygenase (Rubisco)] play an important role in the biochemical fixation of carbon dioxide (Field and Mooney, 1986). The formation of N_2_O in plants is a complex physiological process, including photosynthesis, nitrogen assimilation, and transpiration (Hu et al., 2021). Leaf TN, *T*r, α, *ETR*_*max*_, *I*_*k*_, *Fv/Fm*, *Y*(II), and *SPAD* were all positively correlated with plant N_2_O emissions (Figure 3). The relationship between stomatal conductance, leaf nitrogen content, and nitrogen oxide emission rate can explain the variation of plant capacity to release atmospheric nitrogen oxides (Teklemariam and Sparks, 2006). A correlation between transpiration rates and nitrogen emissions was found in nitrogen compounds released by plants (Stutte et al., 1979). Studies have shown that transpiration rate and other physiological processes affect the transport and emission of N_2_O in plants (Chang et al., 1998; Pihlatie et al., 2005; Borah and Baruah, 2016). The above research results are similar to our results. Leaf nitrogen content, stomatal conductance, and transpiration rate are significantly positively correlated with plant N_2_O emission (Figure 3). Nitrite assimilation in chloroplasts can produce intermediates that react to produce N_2_O. At the same time, there is a negative correlation between N_2_O emission and NO_2_ assimilation (Dean and Harper, 1986; Hakata et al., 2003). Many scholars have discussed the possible sites, mechanisms, and enzymes involved in N_2_O production in plant cells. The mitochondria of plants have a protective mechanism to increase NO scavenging. NADH might act as an electron donor to reduce cytochrome c oxidase (CcO), leading to the conversion of NO to N_2_O (de Oliveira et al., 2008; Gupta et al., 2016; Timilsina et al., 2020). The phenomenon that photosynthesis is closely related to plant N_2_O emission does not only appear in forest ecosystems. In aquatic ecosystems, algae contribute significantly to N_2_O emissions. The green microalga *Chlamydomonas reinhardtii* reduces NO into N_2_O using photosynthetic electron transport and is catalyzed by flavodiiron proteins. The above research provides a new mechanistic understanding of N_2_O production by eukaryotic phototrophs (Burlacot et al., 2020).

Structural equation modelling revealed the process of leaf traits affecting plant N_2_O emissions (Figure 5). Nitrogen and phosphorus in leaves are essential nutrients for carbon assimilation in photosynthesis, which can affect the plant’s photosynthesis (Mo et al., 2019). Photosynthesis is closely related to chlorophyll fluorescence (Mareckova et al., 2019). Leaf area was affected by photosynthesis, and plant N_2_O emission was closely related to leaf area (Figure 5). The formation of plant N_2_O emission is a complex physiological process closely linked with various steps, and the enzymes and pathways involved need to be further studied.

## Conclusion

In conclusion, our study indicates that nitrogen and animal carcass-derived biochar addition affect the functional traits of *C. camphora* seedlings. Nitrogen addition substantially increases plant N_2_O emissions. All seedling biomass was consistently increased by biochar addition, indicating pig carcass biochar will potentially benefit the leaf-harvesting *C. camphora* industry. However, while seedling leaf area was increased by biochar, plant N_2_O emissions were not influenced by biochar. As an important source of atmospheric N_2_O, plant N_2_O emissions deserve more attention. Plant N_2_O emission may be closely related to leaf TN, leaf TP, *P*_*n*_, *C*_*i*_, *T*r, WUE_*L*_, α, *ETR*_*max*_, and *I*_*k*_. Future studies on the mechanisms underlining N and biochar’s effects on plant N_2_O emissions should be conducted, especially in plantations with intensive N fertilization practices.

## Data Availability Statement

The original contributions presented in the study are included in the article/Supplementary Material, further inquiries can be directed to the corresponding author.

## Author Contributions

CZ, HL, and LZ conceived and designed the study. LL, KW, YL, SZ, and SH collected the samples and performed the physiological measurements. XG commented on the manuscript. CZ and HL wrote the manuscript. LZ supervised the whole work. All authors contributed to the article and approved the submitted version.

## Conflict of Interest

The authors declare that the research was conducted in the absence of any commercial or financial relationships that could be construed as a potential conflict of interest.

## Publisher’s Note

All claims expressed in this article are solely those of the authors and do not necessarily represent those of their affiliated organizations, or those of the publisher, the editors and the reviewers. Any product that may be evaluated in this article, or claim that may be made by its manufacturer, is not guaranteed or endorsed by the publisher.

## Supplementary Material

Supplementary Figure 1 Plant height, ground diameter, leaf number, leaf mass, stem mass, root mass, total mass, and root shoot ratio in biochar and control conditions at different nitrogen levels. Significant differences between nitrogen additions are indicated by a small letter (control, BC_0_), or a capital letter (biochar, BC_1_). Tukey *post-hoc* tests were used to determine the significance of the levels of nitrogen. Differences between biochar and control conditions within the same nitrogen levels are indicated with a star, **P* < 0.05; ^**^*P* < 0.01; ^***^*P* < 0.001.

Supplementary Figure 2 Leaf TN, TP, TK, stem TN, TP, TK, root TN, TP, TK, in biochar and control conditions at different nitrogen levels. Significant differences between nitrogen additions are indicated by a small letter (control, BC_0_), or a capital letter (biochar, BC_1_). Tukey *post-hoc* tests were used to determine the significance of the levels of nitrogen. Differences between biochar and control conditions within the same nitrogen levels are indicated with a star, **P* < 0.05; ^**^*P* < 0.01.

Supplementary Figure 3 Photosynthetic gas exchange parameters of *P*_*n*_, *G*_*s*_, *C*_*i*_, *T*r, and WUE_*L*_ in biochar and control conditions at different nitrogen levels. Significant differences between nitrogen additions are indicated by a small letter (control, BC_0_), or a capital letter (biochar, BC_1_). Tukey *post-hoc* tests were used to determine the significance of the levels of nitrogen. Differences between biochar and control conditions within the same nitrogen levels are indicated with a star, **P* < 0.05; ^**^*P* < 0.01; ^***^*P* < 0.001.

Supplementary Figure 4 Chlorophyll fluorescence kinetic parameters of α, *ETR*_*max*_, *I*_*k*_, *Fv/Fm*, and *Y*(II) in biochar and control conditions at different nitrogen levels. Significant differences between nitrogen additions are indicated by a small letter (control, BC_0_), or a capital letter (biochar, BC_1_). Tukey *post-hoc* tests were used to determine the significance of the levels of nitrogen. Differences between biochar and control conditions within the same nitrogen levels are indicated with a star, **P* < 0.05; ^**^*P* < 0.01.

Supplementary Figure 5 Dynamics of leaf area (10^–2^ m^2^) as affected by nitrogen (control, Con; 100 mg N kg^–1^ dry soil, N_1_; 200 mg N kg^–1^ dry soil, N_2_; 300 mg N kg^–1^ dry soil, N_3_), biochar (control, BC_0_; biochar addition, BC_1_) addition over the study. Results of ANOVA with time as random effects are shown. NS, not significant; ^****^*P* < 0.0001.

Supplementary Figure 6 Dynamics of plant N_2_O emission rates as affected by nitrogen (control, Con; 100 mg N kg^–1^ dry soil, N_1_; 200 mg N kg^–1^ dry soil, N_2_; 300 mg N kg^–1^ dry soil, N_3_) or biochar addition (control, BC_0_; biochar addition, BC_1_) over the study. Results of ANOVA with time as random effects are shown. NS, not significant; ^****^*P* < 0.0001.
