# Supplementary material for: Nitrogen and Biochar Addition Affected Plant Traits and Nitrous Oxide Emission From Cinnamomum camphora
Source: Front Plant Sci. 2022 May 10;13:905537. doi: 10.3389/fpls.2022.905537 (PMC9127667; doi:10.3389/fpls.2022.905537)
Supplement: Supplementary file 1 [file Table_1.docx]

**Supplementary table:**

**TABLE S1** Soil and biochar physiochemical properties (n = 4, means with SE). TN, total nitrogen; C, carbon; AP, available phosphorus; TP, total phosphorus; TK, total potassium.

| Soil |  | Biochar |  |
| --- | --- | --- | --- |
| TN (g kg^-1^) | 0.39±0.08 | TN (g kg^-1^) | 26.0±0.75 |
| Organic C (g kg^-1^) | 7.01±0.5 | Organic C (g kg^-1^) | 260.5±12.2 |
| NH_4_^+^-N (mg kg^-1^) | 3.79±0.05 | O (g kg^-1^) | 239.8±16.2 |
| NO_3_^-^-N (mg kg^-1^) | 1.20±0.05 | Ca (g kg^-1^) | 281.5±11.12 |
| TP (g kg^-1^) | 0.38±0.08 | TP (g kg^-1^) | 125.4±6.58 |
| AP (mg kg^-1^) | 5.26±0.23 | TK (g kg^-1^) | 13.15±0.68 |
| pH | 4.99±0.06 | pH | 10.41±0.04 |
