# Supplementary figures and images for: Nitrogen and Biochar Addition Affected Plant Traits and Nitrous Oxide Emission From Cinnamomum camphora
Source: Front Plant Sci. 2022 May 10;13:905537. doi: 10.3389/fpls.2022.905537 (PMC9127667; doi:10.3389/fpls.2022.905537)

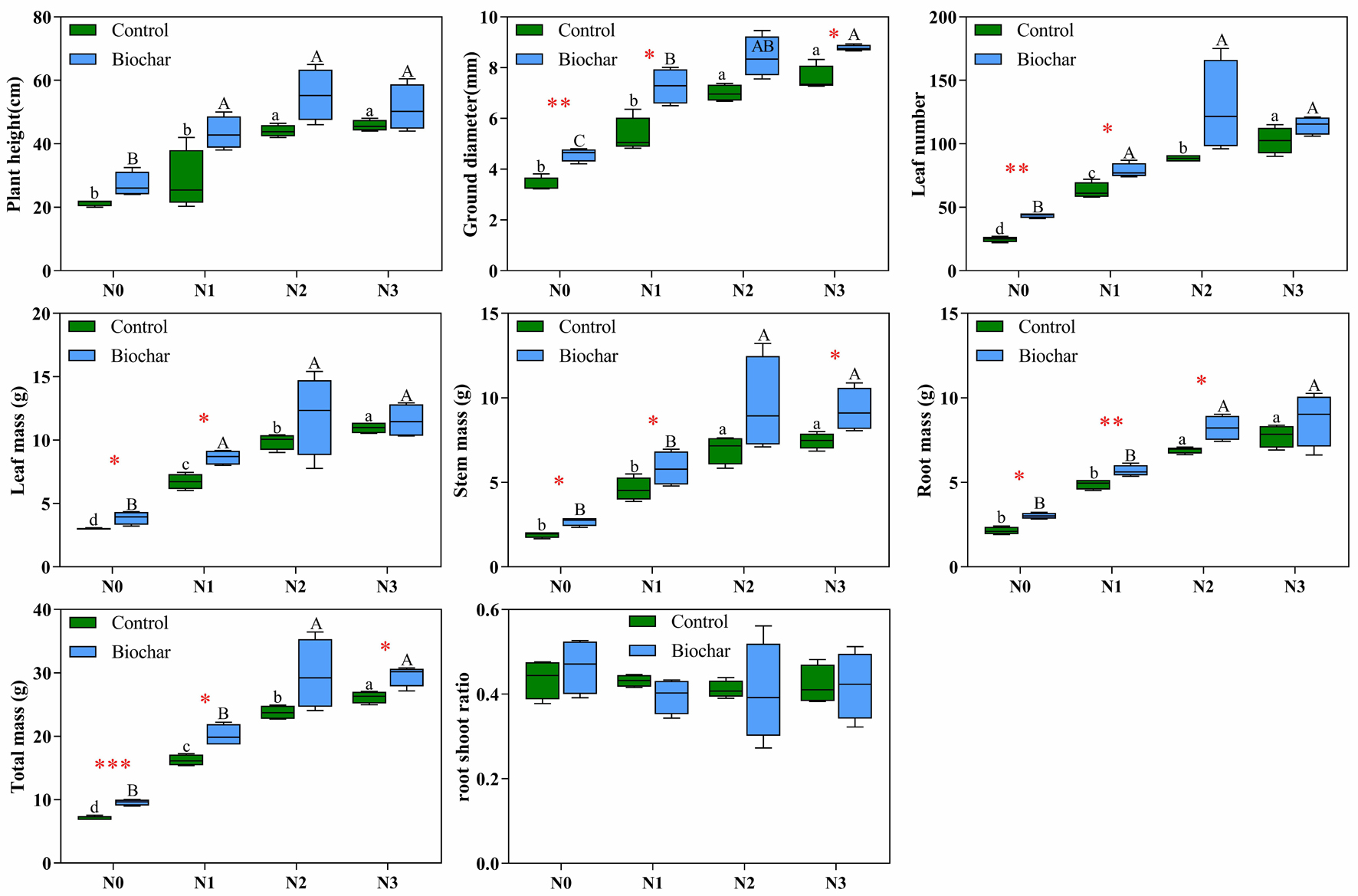

Supplement: Supplementary Figure 1 — Plant height, ground diameter, leaf number, leaf mass, stem mass, root mass, total mass, and root shoot ratio in biochar and control conditions at different nitrogen levels. Significant differences between nitrogen additions are indicated by a small letter (control, BC0), or a capital letter (biochar, BC1). Tukey post-hoc tests were used to determine the significance of the levels of nitrogen. Differences between biochar and control conditions within the same nitrogen levels are indicated with a star, *P < 0.05; **P < 0.01; ***P < 0.001. [file Image_1.JPEG]

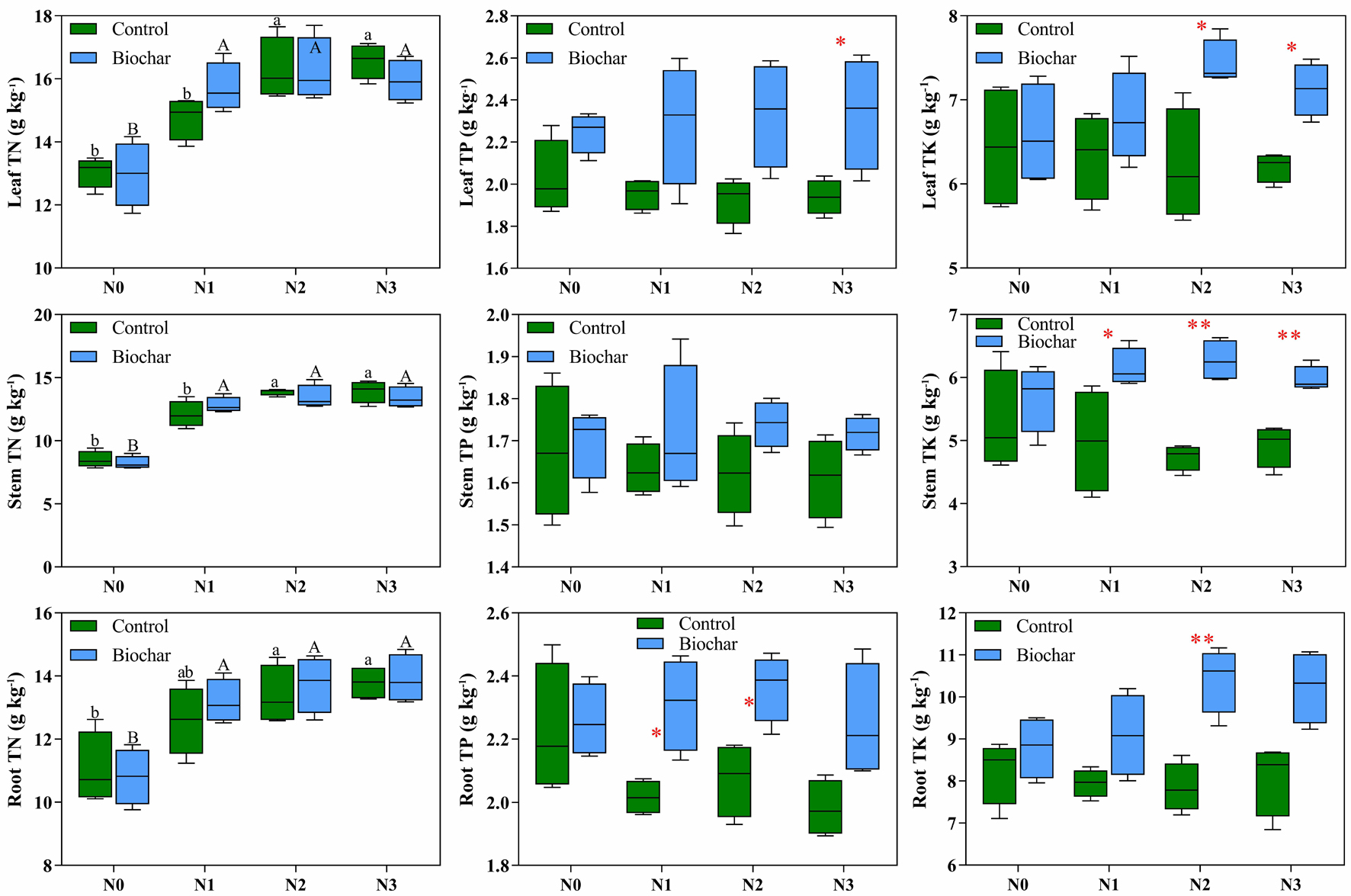

Supplement: Supplementary Figure 2 — Leaf TN, TP, TK, stem TN, TP, TK, root TN, TP, TK, in biochar and control conditions at different nitrogen levels. Significant differences between nitrogen additions are indicated by a small letter (control, BC0), or a capital letter (biochar, BC1). Tukey post-hoc tests were used to determine the significance of the levels of nitrogen. Differences between biochar and control conditions within the same nitrogen levels are indicated with a star, *P < 0.05; **P < 0.01. [file Image_2.JPEG]

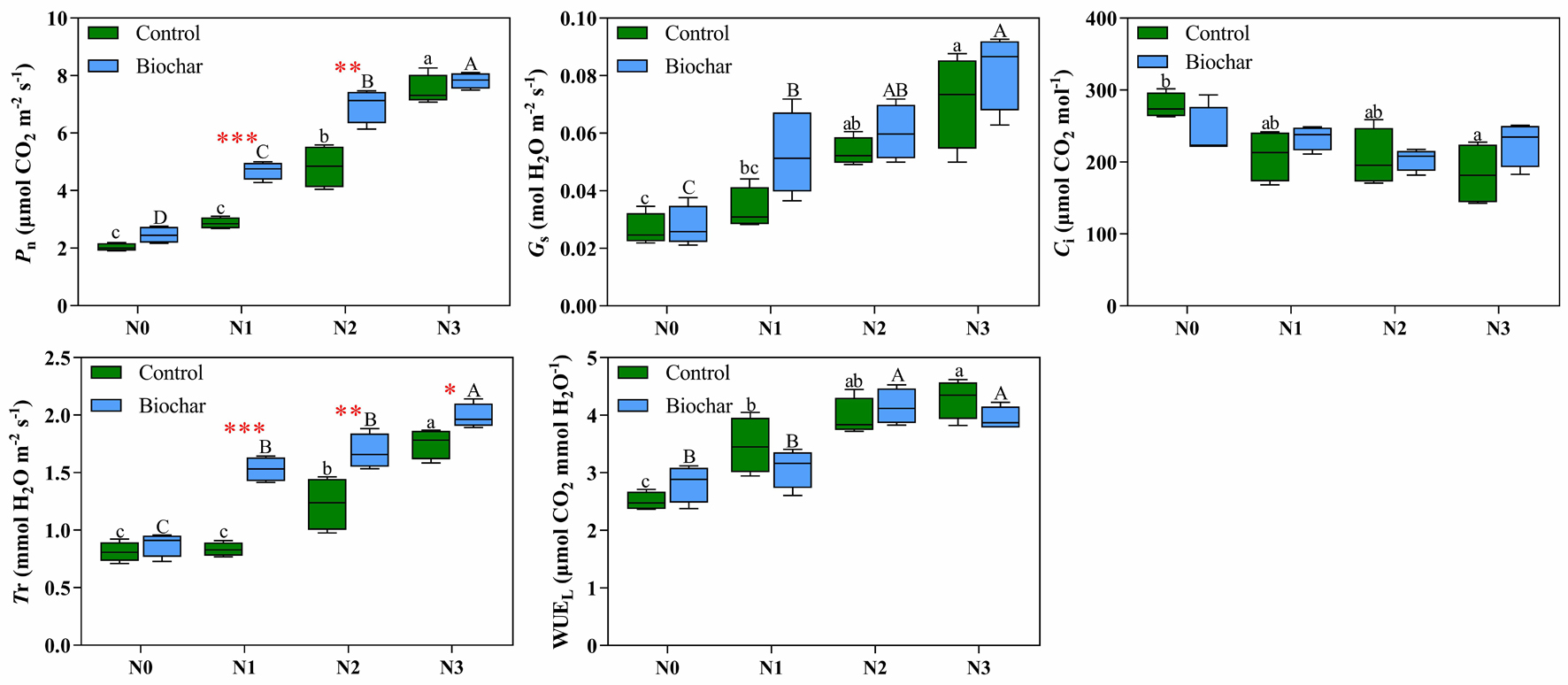

Supplement: Supplementary Figure 3 — Photosynthetic gas exchange parameters of Pn, Gs, Ci, Tr, and WUEL in biochar and control conditions at different nitrogen levels. Significant differences between nitrogen additions are indicated by a small letter (control, BC0), or a capital letter (biochar, BC1). Tukey post-hoc tests were used to determine the significance of the levels of nitrogen. Differences between biochar and control conditions within the same nitrogen levels are indicated with a star, *P < 0.05; **P < 0.01; ***P < 0.001. [file Image_3.JPEG]

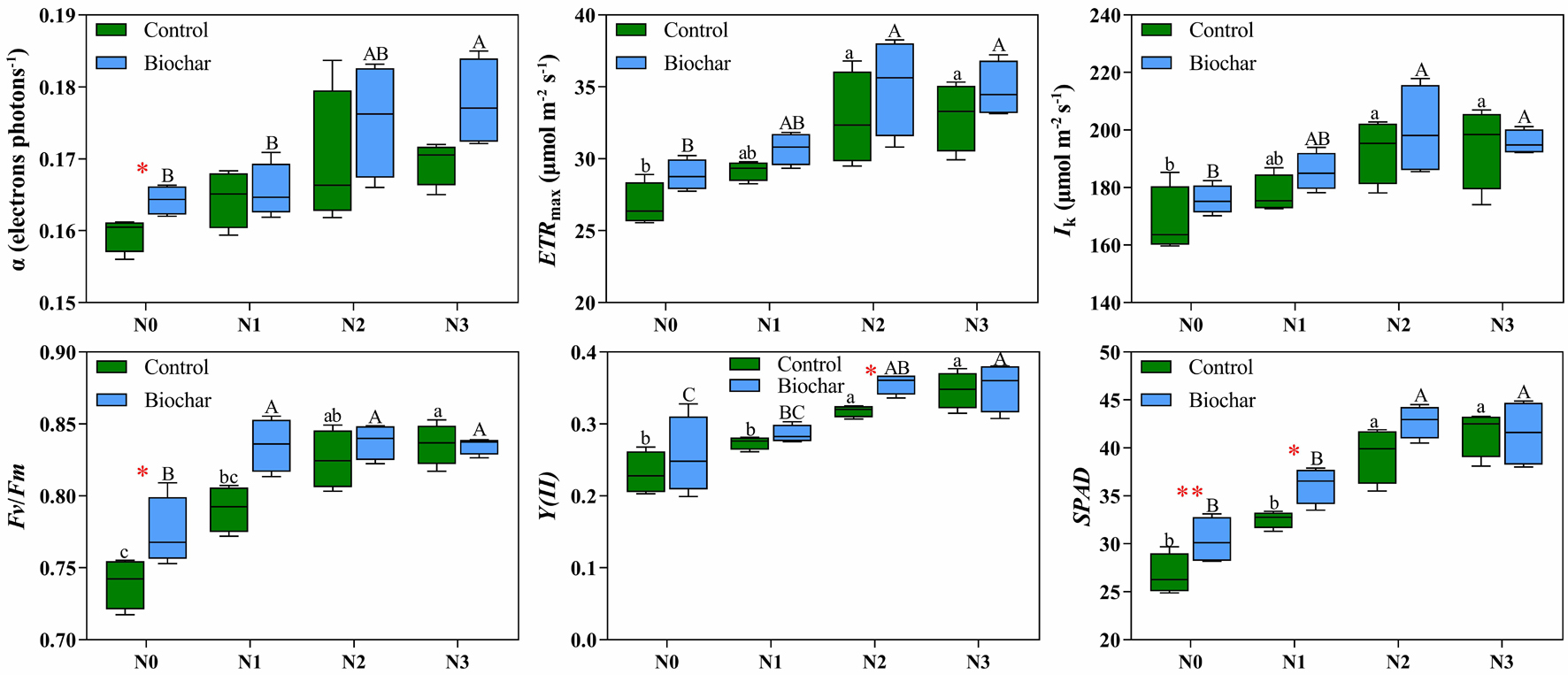

Supplement: Supplementary Figure 4 — Chlorophyll fluorescence kinetic parameters of α, ETRmax, Ik, Fv/Fm, and Y(II) in biochar and control conditions at different nitrogen levels. Significant differences between nitrogen additions are indicated by a small letter (control, BC0), or a capital letter (biochar, BC1). Tukey post-hoc tests were used to determine the significance of the levels of nitrogen. Differences between biochar and control conditions within the same nitrogen levels are indicated with a star, *P < 0.05; **P < 0.01. [file Image_4.JPEG]

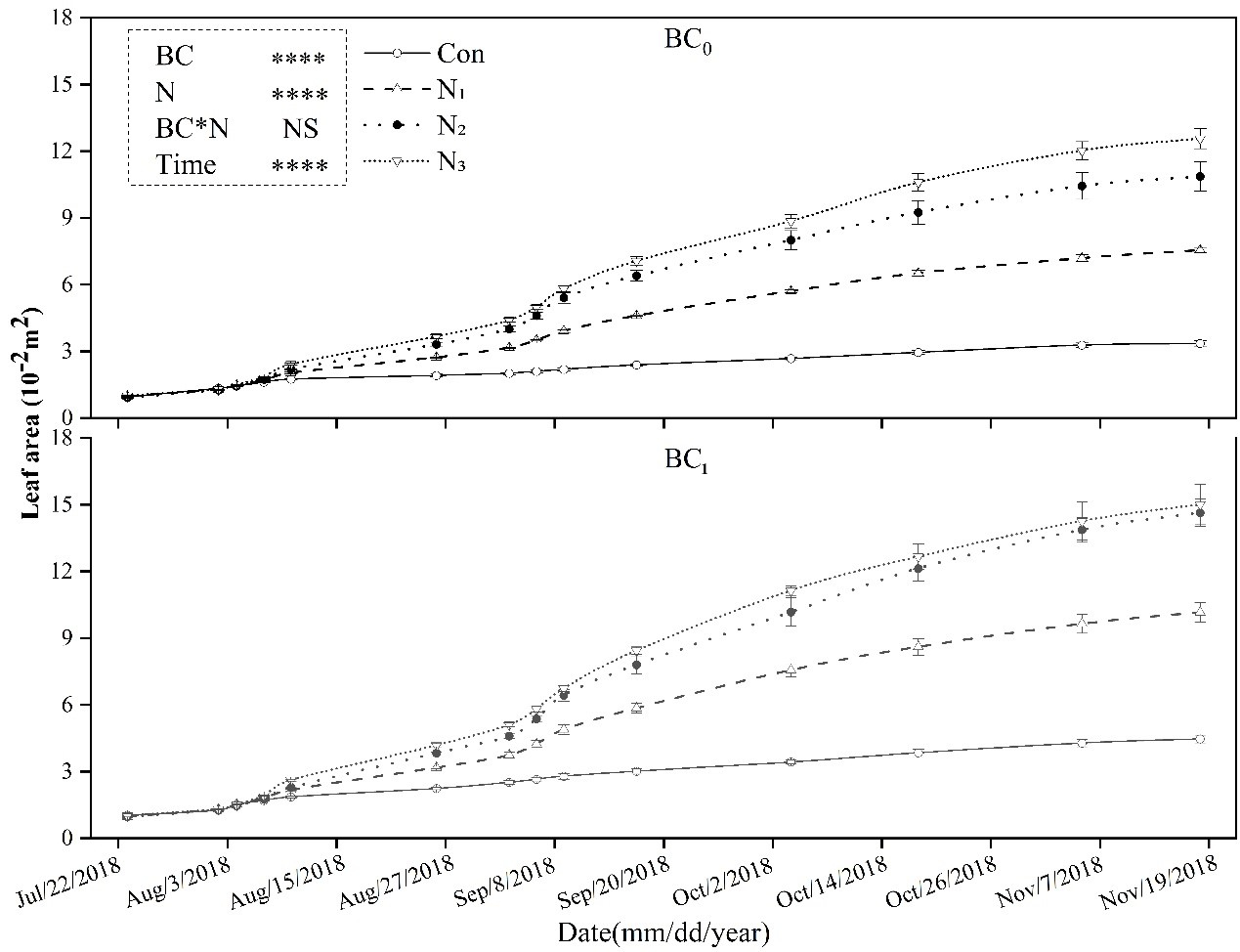

Supplement: Supplementary Figure 5 — Dynamics of leaf area (10–2 m2) as affected by nitrogen (control, Con; 100 mg N kg–1 dry soil, N1; 200 mg N kg–1 dry soil, N2; 300 mg N kg–1 dry soil, N3), biochar (control, BC0; biochar addition, BC1) addition over the study. Results of ANOVA with time as random effects are shown. NS, not significant; ****P < 0.0001. [file Image_5.JPEG]

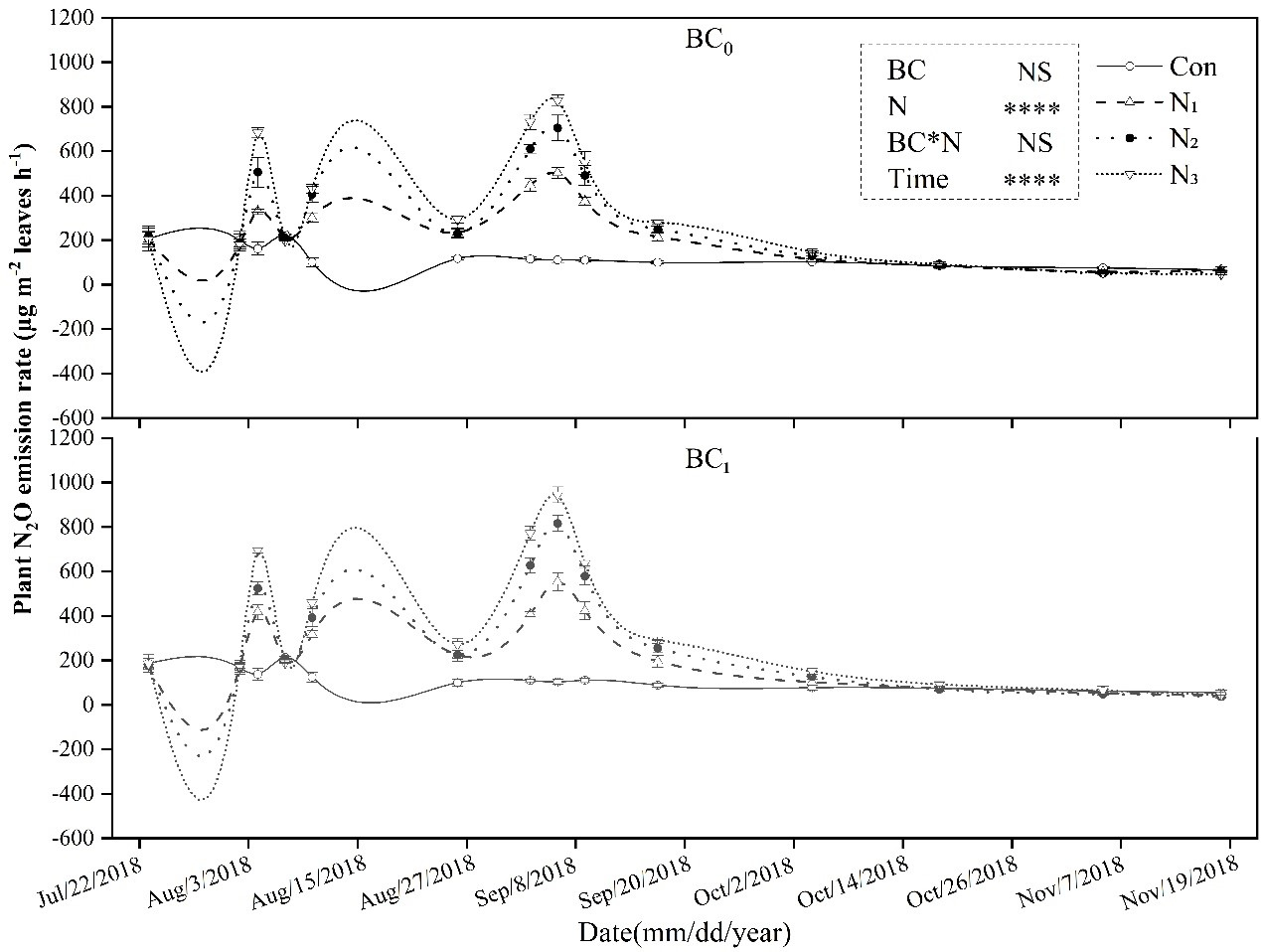

Supplement: Supplementary Figure 6 — Dynamics of plant N2O emission rates as affected by nitrogen (control, Con; 100 mg N kg–1 dry soil, N1; 200 mg N kg–1 dry soil, N2; 300 mg N kg–1 dry soil, N3) or biochar addition (control, BC0; biochar addition, BC1) over the study. Results of ANOVA with time as random effects are shown. NS, not significant; ****P < 0.0001. [file Image_6.JPEG]
